# Effects of the Toxic Non-Protein Amino Acid β-Methylamino-L-Alanine (BMAA) on Intracellular Amino Acid Levels in Neuroblastoma Cells

**DOI:** 10.3390/toxins15110647

**Published:** 2023-11-09

**Authors:** Jake P. Violi, Lisa Pu, Sercan Pravadali-Cekic, David P. Bishop, Connor R. Phillips, Kenneth J. Rodgers

**Affiliations:** 1 School of Life Sciences, Faculty of Science, The University of Technology Sydney, Ultimo, NSW 2007, Australia; j.violi@unsw.edu.au (J.P.V.); lisa.pu@uts.edu.au (L.P.); connor.phillips@uts.edu.au (C.R.P.); 2 School of Mathematical and Physical Sciences, Faculty of Science, The University of Technology Sydney, Ultimo, NSW 2007, Australiadavid.bishop@uts.edu.au (D.P.B.)

**Keywords:** β-methylamino-L-alanine (BMAA), L-serine, amino acid, motor neuron disease, amyotrophic lateral sclerosis

## Abstract

The cyanobacterial non-protein amino acid (AA) β-Methylamino-L-alanine (BMAA) is considered to be a neurotoxin. BMAA caused histopathological changes in brains and spinal cords of primates consistent with some of those seen in early motor neuron disease; however, supplementation with L-serine protected against some of those changes. We examined the impact of BMAA on AA concentrations in human neuroblastoma cells in vitro. Cells were treated with 1000 µM BMAA and intracellular free AA concentrations in treated and control cells were compared at six time-points over a 48 h culture period. BMAA had a profound effect on intracellular AA levels at specific time points but in most cases, AA homeostasis was re-established in the cell. The most heavily impacted amino acid was serine which was depleted in BMAA-treated cells from 9 h onwards. Correction of serine depletion could be a factor in the observation that supplementation with L-serine protects against BMAA toxicity in vitro and in vivo. AAs that could potentially be involved in protection against BMAA-induced oxidation such as histidine, tyrosine, and phenylalanine were depleted in cells at later time points.

## 1. Introduction

Approximately 10% of motor neuron disease (MND) cases are familial and can be attributed to genetic disorders passed from one generation to the next [1], with the remaining 90% of cases considered sporadic and are attributable to a combination of environmental factors, aging, and genetic susceptibility [2,3,4]. The distribution of MND worldwide is mostly uniform; however, in a few specific locations, a significantly higher occurrence of MND has been reported [5,6,7,8,9]. In some cases, these disease clusters might result from exposure to common environmental factors. The ‘MND exposome’ is defined as the accumulation of environmental exposures that increase disease risk and negatively impact the progression [10]. Longinetti and colleagues evaluated epidemiological evidence for a causal relationship between environmental factors and MND [11]. The cluster studies that met the greatest number of Bradford Hill criteria (nine principles used to establish epidemiological evidence of a causal relationship) were β-N-methylamino-L-alanine (BMAA) and exposure to metals and minerals [11]. Using the same approach, Newell and colleagues reported a similar finding and identified, in order of decreasing significance, BMAA, formaldehyde, selenium, and heavy metals [12]. The analysis of 62 population exposure studies implicated the same group of environmental agents (mean odds ratios in parenthesis): BMAA (2.32), formaldehyde (1.54), heavy metals (2.99), manganese (3.85), mercury (2.74), and zinc (2.78) [12].

BMAA is a toxin produced by blue–green algae (cyanobacteria), a phylum of aquatic bacteria known to produce a wide array of toxins that can negatively impact human health [5,13,14,15]. Rapid growth of cyanobacteria in an aquatic environment can result in an algal bloom capable of turning waterways green and increasing toxin levels in the environment. Unlike smoking and exposure to heavy metals or toxic chemicals, which have been in decline over the past few decades, exposure to algal blooms is increasing in line with the incidence of MND [16].

The first link between cyanobacteria and MND was made in the 1950s on the island of Guam where the indigenous people, the Chamorros, were found to have a neurological disease known locally as lytico-bodig. The disease complex later termed ALS/Parkinson’s disease dementia complex (ALS-PDC) is reported to have occurred at 50 to 100 times the rate of ALS/MND elsewhere [17,18]. A unique cultural practice of the Chamorros was to consume flour made from cycad seeds which were later shown to contain the non-protein amino acid BMAA which was synthesised by symbiotic cyanobacteria from the genus *Nostoc* present in the roots of the cycads [19,20,21]. BMAA is produced by a large array of cyanobacteria genera and is also produced by other algal species including the eukaryotic diatoms [22,23]. Human exposure to cyanobacterial toxins occurs more frequently due to climate change, the eutrophication of water bodies, and decreased water flows which is increasing the occurrence of blooms [24,25]. BMAA was identified in the olfactory bulb of individuals living near a lake with regular algal blooms suggesting this as a possible entry point for BMAA into the human brain [26]. The presence of melainabacteria, a non-photosynthetic cyanobacteria, in the human gut microbiome has also been proposed as a potential source of BMAA [27].

Early animal studies that focused on acute rather than chronic BMAA exposure were inconclusive [28]; however, two more recent primate studies have provided compelling evidence of a link between chronic BMAA exposure (140 days) and pathological changes consistent with pre-clinical MND [29,30]. Vervets (Chlorocebus sabaeus) fed BMAA-dosed fruit developed neurofibrillary tangles (NFT) and sparse β-amyloid deposits in the brain [29]. In a follow-up study, oral dosing with BMAA induced MND-type degeneration of the upper and lower motor neurons in vervets [30]. Motor neuron degeneration was demonstrated by TAR DNA-binding protein 43 (TDP-43) proteinopathy in anterior horn cells, reactive astrogliosis, activated microglia, and damage to myelinated axons in the lateral corticospinal tracts [30]. The mechanism through which these changes occurred, however, is unknown. We previously proposed, based on in vitro studies, that BMAA was capable of exchanging for L-serine in protein synthesis [31]. It was subsequently shown that L-serine reduced BMAA-induced ER stress [32,33,34] and decreased the levels of ubiquitin-positive aggregates in cells suggesting that L-serine was capable of reducing the toxicity of BMAA [35]. When examined in vivo, co-administration of L-serine with BMAA also had a significant impact on the neuropathological changes reported in the vervet studies significantly reducing the density of NFT [29], the amount of reactive gliosis, and the number of protein inclusions in motor neurons [29,30]. In both primate studies, however, L-serine did not reduce the amount of free BMAA nor did it decrease the amount of BMAA associated with proteins [29,30].

One area that has seen limited investigation is how BMAA might impact amino acid homeostasis. An untargeted metabolomic study by Engskog et al. [36] detected seven protein amino acids that had significantly changed in abundance in BMAA-treated cells; however, not all protein amino acids were reported to be detected [36]. The present study examines changes in protein amino acid concentrations over time (0 to 48 h) in BMAA-treated human neuroblastoma cells. Intracellular amino acids are extracted and analysed using a validated hydrophilic interaction liquid chromatography (HILIC) triple quadrupole mass spectrometry (TQMS) amino acid method we previously developed [37].

## 2. Results

Multiple reaction monitoring (MRM) transitions were established for BMAA and added to a mass spectrometry method we previously developed describing the use of acetonitrile (ACN) adducts to increase the sensitivity of amino acid analysis [37]. When optimising detection parameters for BMAA, an ACN adduct, [M + H + ACN]^+^, was also observed but only at the highest standard concentration used (1000 ng/mL) and was at a low intensity. The use of the ACN adduct, like those of the other basic amino acids in this HILIC-TQMS method [37], did not improve the signal-to-noise ratio for BMAA, thus the protonated form of BMAA was used for MRM development instead. Cells were treated with 1000 µM BMAA and intracellular concentrations of BMAA were examined at time points between 3 and 48 h.

**Figure 1:**
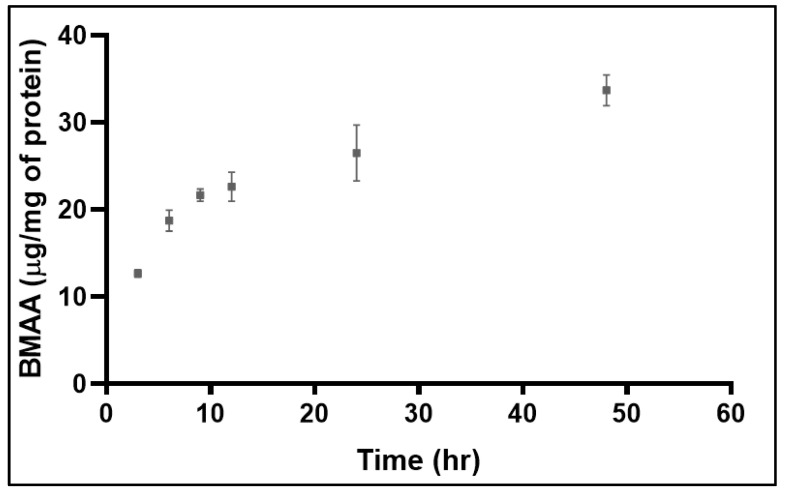
Concentration of intracellular BMAA over 48 h in SH-SY5Y cells incubated with BMAA (1000 µM); error bars represent standard error of the mean (*n* = 6).

Intracellular BMAA was detected in BMAA-treated cells at every time point examined (Figure 1) but no BMAA was detected in untreated cells. The intracellular BMAA concentration in SH-SY5Y cells initially increased rapidly but started to plateau at around 9 h (Figure 1).

No methionine was detected in any cell lysate sample, consistent with our previous findings [37]. At each time point, the percentage change in the concentration of protein amino acids was determined by comparing concentrations in cells exposed to BMAA to those in cells not exposed to BMAA (control cells) (Table 1). Two points of normalisation were employed to ensure that any changes observed were valid. Firstly, the inclusion of the internal standard (ISTD) norvaline accounted for variability in the extraction process or instrument performance while normalisation to total cell protein ensured that changes in amino acid levels were not due to variations in cell number.

Although intracellular amino acid concentrations did not vary significantly between control and BMAA-treated cells at the 0 h time-point, as early as 3 h (the first time point examined), concentrations of eight amino acids were significantly increased and concentrations of two amino acids significantly decreased. By the 6 h time-point, however, all 19 protein amino acids detected had returned to basal levels (Table 1). The most significant changes to amino acid concentrations were observed after 9 h which coincided with the time where BMAA concentrations in the cell had started to plateau (Figure 1). Intracellular concentrations of 15 of the 19 amino acids were significantly decreased relative to control cells, but leucine, tryptophan, cysteine, and glycine were unchanged (Table 1). As was observed previously, this change was transient and all amino acids with the exception of serine had returned to control levels by 12 h. At the 12 and 24 h time-points, all amino acids were maintained at basal concentrations with the exception of histidine which was significantly lower at 24 h and serine which was significantly decreased for the remainder of the culture period. At the 48 h time point, concentrations of nine amino acids were significantly decreased relative to control cells (Table 1). The amino acids significantly decreased were alanine, isoleucine, histidine, phenylalanine, serine, valine, tyrosine, aspartate, and asparagine, all of which had been significantly depleted at the 9 h time point. Arginine, glutamine, glutamic acid, and threonine, which had been significantly decreased at the 9 h time point, were also decreased but the change did not reach statistical significance (Table 1).

The greatest change in an amino acid observed in BMAA-treated cells was that of glycine which increased to 12 fold the concentration in control cells after 3 h but had returned to control cell concentrations by 6 h and did not differ significantly from the control for the remainder of the 48 h culture period (Table 1). The greatest decrease was seen in asparagine which decreased to 23% of the control concentrations at 9 h. No changes were observed in cysteine, leucine, or tryptophan in BMAA-treated cells at any of the time points examined but all the remaining protein amino acids showed statistically significant changes in at least one time point (Table 1). For 2 of the 16 amino acids examined there was a significant change in concentration only at one time point (glycine and proline). Asparagine, glutamine, histidine, isoleucine, phenylalanine, proline, serine, and tyrosine showed a decrease in concentration following BMAA treatment. Seven out of eight these amino acids decreased in two or more time points with only proline decreasing at one time point (9 h). Only glycine showed a significant increase at a single time-point (3 h).

The biggest overall impact on the concentration of an amino acid following incubation with BMAA was observed for serine. Serine was the only amino acid to show significant decreases in concentration at four time points (9, 12, 24, and 48 h) with the largest fall in concentration at the 9 h time point (27% of that in control cells). So, following 9 h of incubation with BMAA, cells were deficient in serine for the remainder of the 48 h.

## 3. Discussion

The rate of uptake of BMAA by SH-SY5Y cells was similar to what has previously been reported for radiolabelled BMAA [31]. After 3 h, when concentrations of BMAA were starting to increase in the treated cells, the concentrations of many protein amino acids were significantly altered (predominantly increased) relative to those in the control cells. By 6 h, however, all amino acids had returned to control concentrations, suggesting that amino acid homeostasis, disturbed by the presence of BMAA, had been re-established in the cell. BMAA had the biggest impact on amino acid concentrations after 9 h, corresponding to the time when BMAA concentrations in the cell were beginning to plateau. Concentrations of 15 of the 19 amino acids detected were significantly decreased after 9 h but by 12 h, basal levels had again been re-established for all amino acids with the exception of serine which was depleted throughout the remaining 48 h culture period.

Intracellular amino acid concentrations are normally maintained by influx from the extracellular environment, biosynthesis, and protein recycling to ensure that free amino acids are sufficient to allow anabolic processes such as protein translation and energy production [38]. Global amino acid deficiencies, as was observed following 9 h exposure to BMAA, can be corrected by increasing autophagic breakdown of proteins through GCN2 signaling [38]. The depletion of protein amino acids results in an increase in uncharged transfer RNAs which activate the serine/threonine kinase GCN2 which then phosphorylates eukaryotic initiation factor 2α (eIF2α) resulting in inhibition of global protein translation [38]. To correct an amino acid deficiency, the translation of mRNAs encoding amino acid biosynthesis regulators, amino acid transporters, and autophagy mediators increases, thus restoring amino acid levels [39]. In certain circumstances, however, the proteasome can supply amino acids to sustain protein synthesis prior to up-regulation of the autophagosomal–lysosomal pathway [40]. Amino acids required for the synthesis of new proteins are supplied by proteasomal degradation of pre-existing proteins; however, nascent and newly formed polypeptides are largely protected from proteolysis [40].

Serine was the amino acid most heavily impacted by BMAA, with concentrations falling to 27% of those in untreated cells at 9 h and then returning to between 60 and 68% of control concentrations for the remainder of the 48 h culture period. The potential clinical impact of BMAA-induced serine deficiency is not known but our data are consistent with studies showing that co-treatment with L-serine protects against BMAA toxicity in vitro [31,41] and can reduce BMAA-induced protein aggregation [35]. L-serine was also protective in a vervet model of pre-clinical MND [29,30] where co-administration of L-serine with BMAA decreased the number of NFTs in upper motor neurons and reduced the number of anterior horn neuron protein inclusions, microglial activation, and reactive astrogliosis in primates [29,30]. It was previously shown that the addition of L-serine to culture medium containing radiolabelled BMAA reduced the amount of radiolabel in the ‘protein fraction’ of a cell lysate in vitro [31]. The protein fraction was the material in the lysate that was insoluble in 10% trichloroacetic acid (TCA) so could potentially contain other macromolecules. Using a cell-free in vitro protein synthesis system, Glover reported that BMAA incorporates as an error in synthesis and could exchange for alanine and serine in proteins [42]. However, studies examining amino acid activation using ATP/PPi exchange assays with recombinant human seryl tRNA synthetase (SerRS) and alanyl tRNA synthetase (AlaRS) suggested that BMAA was not a substrate for SerRS but was a weak substrate for AlaRS aminoacylation and could escape the intrinsic proofreading mechanism so could potentially become misincorporated into proteins in place of alanine [43]. BMAA was found in the protein fraction in plasma and brain extracts in a primate study; however, concentrations were not reduced following dietary supplementation with L-serine despite a positive effect on pathological changes [29,30]. These data are consistent with exogenous L-serine correcting a deficiency in L-serine caused by BMAA.

Serine is a non-essential amino acid and since the culture medium employed in the present studies did not contain serine (Table S2), cells were reliant on the serine biosynthesis pathway (SBP) in which serine is synthesised from the glycolytic intermediate 3-phosphoglycerate in a three-step process [44]. The enzyme phosphoglycerate dehydrogenase (PHGDH) catalyses the committed step in de novo serine biosynthesis [44]. The SBP provides precursors for biosynthesis of proteins, nucleotides, phospholipids, and glutathione [45]. The decrease in intracellular serine concentrations could have resulted from either a decrease in serine synthesis or an increased consumption of serine. Serine starvation in vitro activates de novo serine synthesis, diverting glycolytic intermediates away from the tricarboxylic acid (TCA) cycle and reducing the amount of oxaloacetate entering the cycle [45]; in response, cells increase oxidative phosphorylation to maintain ATP turnover [46]. Serum metabolic profiling revealed that metabolites associated with changes in energy metabolism and amino acid metabolism were the most significantly altered in neonatal rats exposed to BMAA [47].

At 48 h, levels of the amino acids asparagine and aspartate were significantly decreased. Asparagine can be hydrolysed to aspartate which undergoes transamination to form glutamate and oxaloacetate which provides fuel for the TCA cycle [48]. Serine biosynthesis through PHGDH also plays an important role in maintaining folate pools for nucleotide synthesis in dividing cells and by contributing glycine to the backbone of purines [49]. It is possible that a sustained decrease in serine levels in neuronal cells could contribute to the neurotoxic effects reported for BMAA as L-serine plays an important role in the CNS and is the metabolic precursor of the neuroactive substances, D-serine, and glycine [44]. L-serine also serves as a building block for phosphatidylserine and sphingolipids which are important components of the plasma membrane [44]. A deficiency in L-serine can result in the synthesis of neurotoxic deoxysphingolipids and lipid body deposition in cells [50]. Humans with mutated PHGDH have lower levels of free L-serine in the plasma and in cerebrospinal fluid and exhibit severe neurological symptoms, including congenital microcephaly, psychomotor retardation, and intractable seizures [51,52]. PHGDH knock-out mice display severe consequences of embryonic development, such as brain malformation with overall growth retardation, and die after embryonic day 13.5 [53].

Glycine showed the biggest single change in concentration with a spike at the 3-h time point reaching 12-fold concentrations in control cells but, by 6 h, returned to basal levels which were maintained for the remainder of the 48 h. Glycine is a non-essential amino acid; it was not present in the culture medium but can be synthesised from threonine, serine, and choline [54]. Glycine plays a role in many key reactions in the cell such as gluconeogenesis and is used in the formation of important biological molecules such as glutathione, purines, and creatine [54]. High concentrations of glycine trigger the rapid removal of glycine via the glycine cleavage system [55].

It has been suggested that alterations to the neuronal histaminergic system may play a role in the pathophysiology of several neurological disorders including Parkinson’s and Alzheimer’s disease [56,57], with post-mortem studies showing reduced histamine in several regions of the brain [58]. Histidine is normally obtained from the diet and was supplied in the culture medium in the present studies (Table S2). Levels of histidine were lower in BMAA-exposed cells than in control cells from 9, 24, and 48 h. The reactivity of the imidazole ring confers a pH buffering capacity, the ability to chelate metals, and the ability to scavenge reactive oxygen and nitrogen species on the molecule [59]. Histidine turnover occurs mainly through protein synthesis and its major route of catabolism is via urocanate to glutamate [59]. In the present studies, histidine levels in BMAA-treated cells were around 25% lower than those in control cells after 9 h, raising the possibility that it could have been consumed in protecting cells against oxidative stress. BMAA has been shown to induce oxidative stress and this has been suggested to be integral to its neurotoxicity [60,61]. Other important targets of oxidation in living systems are the phenolic amino acids tyrosine and phenylalanine [62,63]. Loss of these amino acids can result from oxidative stress mediated by reactive oxygen and nitrogen species [63]. In the present studies, there were intermittent drops in tyrosine levels and to a lesser extent in phenylalanine levels which could reflect the modification of these native amino acids by reactive species and the subsequent replenishment of the amino acids from the medium that contained phenylalanine and tyrosine (Table S2).

Little is known about the impact that non-protein amino acids have on protein amino acid homeostasis and no previous studies have specifically examined the effects of BMAA on amino acid levels in vitro. A metabolomic study by Engskog and colleagues [36] using differentiated SH-SY5Y cells exposed to BMAA for 24 h reported changes in certain amino acid levels. Enkskog reported that concentrations of seven amino acids were increased at 24 h (leucine, phenylalanine, glutamine, isoleucine, proline, threonine, and tryptophan) in the non-dividing cells while the concentration of lysine was decreased and the remaining protein amino acids not reported to change or possibly not detected. As was demonstrated in the present study, amino acid concentrations change over time; examining concentrations at a single time-point might not reveal the broader impact of BMAA on amino acid metabolism.

In the present studies, individual protein amino acid concentrations varied greatly in both treated and control cells (Figures S2–S17). The average intracellular amino acid concentration at the 3 h time point was 11 µg/mg protein and at the 12 h time-point was 20 µg/mg protein (Figures S2–S17). The intracellular BMAA concentration was 12 µg/mg protein at 3 h and 22 µg/mg protein at 12 h (Figure 1) so was comparable to that of protein amino acids. While these studies examined the impact of BMAA on protein amino acid homeostasis in vitro, it is not known if comparable intracellular BMAA concentrations could exist in vivo or if chronic low level of exposure to BMAA could similarly impact amino acid homeostasis. An additional factor that could also be considered is if ‘protein-associated’ BMAA could provide a reservoir and maintain intracellular concentrations of BMAA [64].

## 4. Conclusions

BMAA had a profound effect on intracellular free amino acid concentrations in human neuroblastoma cells over a 48 h culture period. In most cases, homeostasis was re-established and intracellular protein amino acids returned to control concentrations. A key finding was the depletion of serine in BMAA-treated cells after 9 h which was not corrected and this could have implications for global protein synthesis but could also impact energy generation in the cell through diverting glycolytic intermediates for the TCA cycle into the serine biosynthetic pathway. In addition, a deficiency in serine can promote the synthesis of neurotoxic deoxysphingolipids [50]. Correction of serine depletion could also be a factor in the observation that supplementation with L-serine is protective against BMAA toxicity in vitro [31,41] and in vivo. [29,30]. Other amino acids that could potentially be involved in protection against BMAA-induced oxidation such as histidine, tyrosine, and phenylalanine were depleted in cells at later time points.

## 5. Materials and Methods

### 5.1. Cell Culture and Treatment

The SH-SY5Y (human neuroblastoma) cell line was obtained from The American Type Culture Collection (ATCC) (Manassas, VA, USA). Cells at passage 20 were seeded at 125,000 cells per well into 12-well plates in Eagle’s minimal essential medium (EMEM) (Table S2) supplemented with 5% glutamax and 10% fetal bovine serum (FBS) and left for 24 h. In each plate, 6 wells were designated as control cells and 6 wells as BMAA-treated cells. Media was removed and then replaced with either EMEM with 5% glutamax and 10% FBS (control) or the same medium containing 1000 µM BMAA (BMAA-treated). At 0 h, 3 h, 6 h, 9 h, 12 h, 24 h, and 48 h, the media was removed and each well was washed three times with warmed phosphate buffered saline (PBS). Plates were then snap frozen with liquid nitrogen and stored at −80 °C prior to amino acid extraction. At the time point when cells are harvested, a protein assay was performed on cell lysates from each well of treated and control cultures (Section 5.3). This allowed amino acid concentrations to be normalised to protein (and expressed as µg/mg protein), allowing for any variation in cell numbers in each well. In addition, it provided a surrogate measure of cell numbers throughout the culture period as an indication of cell death. There was no significance difference between control cell protein concentrations (average values; 46.4 mg/mL, standard deviation 2.2) and treated cell protein concentrations (average values; 48.6 mg/mL, standard deviation 6.0) at any of the time points. Examination of cell morphology and density in each well using an inverted phase contrast microscope could not identify any differences between the treated and control cells.

### 5.2. Amino Acid Extraction

In total, 500 μL of a 500 ng/mL solution of the internal standard (ISTD) norvaline was prepared in ultrapure water and added to each well of the 12 well plate. Cells were removed using a cell scraper and the cell suspension transferred to a 2 mL tube. An additional 400 μL of ultrapure water was added to each well to recover any remaining cells and then added to the tube. Trichloroacetic acid (TCA) (Sigma-Aldrich, Castle Hill, NSW, Sydney, Australia, made with ultrapure water) was added to give a final concentration of 10% (*w*/*v*). Each sample was then subjected to probe sonication (Qsonica Q125 Sonicator Thermo Fisher Scientific, Scoresby, VIC, Australia) for 30 s at 50% power; this was repeated twice to ensure complete cell lysis, with the samples left on ice for 1 min between repeats. Samples were then centrifuged at 15,000 g for 15 min at 4 °C and the supernatant removed and transferred to new tube. Pellets were washed with 200 μL of cold 10% (*w*/*v*) TCA, vortexed, and centrifuged again with the supernatant collected and transferred to the corresponding 2 mL tube; this was then repeated to allow for triplicate washes of the pellet resulting in a final volume for the free amino acid containing tubes being 1.4 mL. In total, 200 μL of 0.1% (*w*/*v*) triton X-100 (Sigma-Aldrich, Castle Hill, NSW, Sydney, Australia) was added to the remaining pellets and the samples were then vortexed and 200 μL RIPA buffer (Thermo Fisher Scientific, Scoresby, VIC, Australia) was also added to allow for further protein solubilisation. The free amino acid-containing samples were then sublimated by freeze-drying (Martin Christ, alpha 2-4 LD plus) at 0.1 mbar and −80 °C for 16 h. Dried samples were reconstituted in 150 μL of 20 mM HCl with 10 mM DTT and spun through a 0.22 μm membrane filter (Ultrafree-MC LG Centrifugal 0.2 μm pore size PTFE Membrane Filter (UFC30LG25) Sigma-Aldrich, Macquarie Park, NSW, Australia) for 10 min at 5000× *g*. Samples were stored at −80 °C until LC-MS/MS analysis. Prior to analysis, the samples were diluted 1:10 with acetonitrile (ACN) to match the initial chromatographic mobile phase conditions (90:10 ACN:H_2_O).

Amino acid concentrations in each sample were normalised to the norvaline signal in the sample. The ISTD (norvaline) was added at the beginning of the analyte extraction procedure allowing any loss during sample preparation to be accounted for. The deviation in the ISTD response in the MS from that of the norvaline standard allowed an adjustment factor to be determined and applied to each amino acid, thus normalising amino acid concentrations in that sample to the ISTD.

### 5.3. Protein Assay

Solution of 4% (*w*/*v*) copper(II) sulphate (CuSO_4_) was diluted 1 in 50 with bicinchoninic acid (BCA) solution (Sigma-Aldrich, Castle Hill, NSW, Australia). In total, 10 μL of each sample was added to the wells of a series of 96 well-plates; this was performed in triplicate for each sample. An 8-point calibration curve was constructed of bovine serum albumin (BSA) in 0.05% (*w*/*v*) triton X-100 and 50% RIPA buffer, with 10 μL of each also being loaded into a well a 96 well-plate; this was also performed in triplicate. Overall, 100 μL of the CuSO_4_ BCA solution was added to each well each of the 96 well plates. The colour was left to develop for 2 h at room temperature. Each of the 96 well-plates were then read in a Tecan Infinite M1000 PRO monochromator microplate reader. The absorbance of each well was read at a wavelength of 562 nm with the number of flashes being set to 25.

### 5.4. HILIC-TQMS

An amino acid HILIC-TQMS method was previously developed [37] and multiple reaction monitoring (MRM) transitions for BMAA were added to the other 20 amino acids (see supplementary data Table S1 for transitions). BMAA was subjected to the same validation metrics used to validate the other amino acids in the original method. BMAA had a linear range of 5 ng/mL to 1000 ng/mL, an LOD of 0.38 ng/mL, an LOQ of 1.17 ng/mL, and exhibited good repeatability with a percentage relative standard deviation (%RSD) calculated from seven repeat injections of the same standard to be less than 10%. HILIC-TQMS was conducted on a Shimadzu Nexra X2 UHPLC coupled to a Shimadzu 8060 triple quadrupole mass spectrometer with a Waters BEH Amide column (2.1 mm × 100 mm, 1.7 μm particle size). The flow rate was 0.8 mL/min with a column oven temperature of 30 °C. Amino acids were eluted with a stepped gradient; solvent A consisted of 80 mM ammonium formate in ultrapure water + 0.6% formic acid (FA) and solvent B consisted of acetonitrile (ACN) + 0.6% FA with the following chromatographic compositions of solvent B: 0.00 min 90%, 3.50 min 90%, 5.50 min 80%, 9.25 min 80%, 9.30 min 70%, 11.20 min 70%, 11.20 min 90%, and 14.00 min 90% (separation of all amino acids is shown in Figure S1). The TQMS was run in positive mode with the following source parameters: 0.1 kV interface voltage, 400 °C interface temperature, 225 °C desolation line (DL) temperature and 400 °C heat block, 3 L/min nebulising gas flow, 17 L/min heating gas flow, and 3 L/min drying gas flow. A 10-point calibration curve (0.1 ng/mL, 1 ng/mL, 5 ng/mL, 10 ng/mL, 25 ng/mL, 50 ng/mL, 100 ng/mL, 250 ng/mL, 500 ng/mL, and 1000 ng/mL) of all amino acid standards [37], with the inclusion of BMAA (Sigma-Aldrich, Castle Hill, NSW, Australia) were analysed alongside samples; the injection volume was set to 5 μL with every sample and standard being injected in triplicate. The needle wash contained 1:1:1:1 acetonitrile:methanol:isopropanol:water and was set to external wash only since internal washing could cause retention time shifts for the analytes. No carry over was observed from any samples or from the highest concentration standard (1000 ng/mL) used as we have previously published [37].

### 5.5. Statistical Analysis

The mass spectrometry results were quantified and then subjected to normalisation to both the internal standard and the corresponding protein assay results. A Welch’s *T* test was preformed to determine significant differences between treated and control cells and the results displayed as a percentage of the control values calculated at each time point for each amino acid as described previously [37].

## Figures and Tables

**Table 1:** Amino acid changes shown as a percentage of the corresponding control sample; error shown is standard error of the mean. * denotes a *p* value of <0.05, ** denotes a *p* value of <0.005, and *** denotes a *p* value of <0.0005. Significant increases are highlighted in green and significant decreases are highlighted in red. ND stands for not detected.

Time (h)	Ala	Arg	Asn	Asp	Cys	Glu	Gln	Gly	His	Ile	leu	Lys	Phe	Pro	Ser	Thr	Trp	Tyr	Val
0	130 ± 6.6	110 ± 3.5	140 ± 11	110 ± 3.9	ND	110 ± 3.6	100 ± 3.0	130 ± 8.3	120 ± 10	100 ± 4.4	100 ± 2.1	120 ± 3.3	110 ± 3.2	110 ± 3.2	100 ± 10	120 ± 4.2	100 ± 3.0	140 ± 10	110 ± 4.3
3	230 ± 12 ***	140 ± 3.9 **	120 ± 13	140 ± 3.7 ***	ND	150 ± 4.0 ***	84 ± 2.2 *	1200 ± 260 **	89 ± 5.7	91 ± 2.9	92 ± 2.2	140 ± 3.0 **	100 ± 2.6	97 ± 1.9	120 ± 9.3	160 ± 7.7 *	94 ± 6.1	59 ± 3.5 *	180 ± 8.5 *
6	95 ± 5.4	130 ± 8.1	84 ± 8.0	93 ± 4.3	110 ± 5.2	91 ± 4.2	93 ± 4.7	89 ± 6.9	110 ± 4.8	110 ± 4.8	99 ± 4.2	130 ± 5.8	91 ± 3.9	95 ± 4.0	100 ± 9.2	83 ± 5.9	95 ± 3.3	110 ± 9.1	110 ± 6.0
9	62 ± 4.5 *	74 ± 4.0 *	23 ± 3.8 *	70 ± 4.2 *	88 ± 3.6	73 ± 4.2 *	65 ± 4.3 *	63 ± 4.3	74 ± 2.5 *	48 ± 3.9 **	81 ± 3.2	83 ± 2.8 *	66 ± 5.8 *	74 ± 3.9 *	27 ± 3.7 **	53 ± 2.4 *	90 ± 2.2	59 ± 3.9 **	62 ± 1.7 **
12	97 ± 4.9	110 ± 7.2	95 ± 6.0	100 ± 4.6	100 ± 6.2	110 ± 5.7	110 ± 6.7	110 ± 6.9	85 ± 5.5	88 ± 5.0	86 ± 3.7	100 ± 6.2	88 ± 4.4	110 ± 6.4	66 ± 3.8 *	100 ± 5.3	100 ± 8.3	100 ± 5.7	93 ± 4.6
24	95 ± 6.1	80 ± 5.8	75 ± 6.0	100 ± 7.1	100 ± 7.8	100 ± 7.7	88 ± 6.1	100 ± 6.3	74 ± 4.3 *	91 ± 7.2	86 ± 6.9	88 ± 5.7	91 ± 7.1	92 ± 7.0	62 ± 5.2 *	90 ± 5.9	100 ± 5.3	77 ± 5.8	81 ± 6.1
48	73 ± 5.2 *	76 ± 4.4	59 ± 3.1 *	70 ± 4.1 *	110 ± 8.3	87 ± 7.4	86 ± 8.7	110 ± 17	74 ± 3.6 *	66 ± 4.0 *	82 ± 3.7	85 ± 4.3	71 ± 5.2 *	80.0 ± 3.9	60 ± 4.5 *	81 ± 4.3	100 ± 8.0	69 ± 3.1 *	74 ± 3.5 *

## Data Availability

Data is contained within the article and supplementary material.

## Supplementary Materials

The following supporting information can be downloaded at https://www.mdpi.com/article/10.3390/toxins15110647/s1, Table S1: Limit of Detection (LOD), Limit of Quantification (LOQ) and Multiple Reaction Monitoring (MRM) transitions for all amino acids analysed in this study. Table S2: Amino acid content of the culture medium used in the studies. Figure S1: Total ion chromatograms (TIC) for each amino acid analysed. Figure S2: Intracellular concentrations of alanine for control and BMAA-treated cells. Figure S3: Intracellular concentrations of arginine for control and BMAA-treated cells. Figure S4: Intracellular concentrations of asparagine for control and BMAA-treated cells. Figure S5: Intracellular concentrations of aspartic acid for control and BMAA-treated cells. Figure S6: Intracellular concentrations of cysteine for control and BMAA-treated cells. Figure S7: Intracellular concentrations of glutamic acid for control and BMAA-treated cells. Figure S8: Intracellular concentrations of glutamine for control and BMAA-treated cells. Figure S9: Intracellular concentrations of glycine for control and BMAA-treated cells. Figure S10: Intracellular concentrations of isoleucine for control and BMAA-treated cells. Figure S11: Intracellular concentrations of leucine for control and BMAA-treated cells. Figure S12: Intracellular concentrations of lysine for control and BMAA-treated cells. Figure S13: Intracellular concentrations of phenylalanine for control and BMAA-treated cells. Figure S14: Intracellular concentrations of proline for control and BMAA-treated cells. Figure S15: Intracellular concentrations of threonine for control and BMAA-treated cells. Figure S16: Intracellular concentrations of tryptophan for control and BMAA-treated cells. Figure S17: Intracellular concentrations of valine for control and BMAA-treated cells.
