# Supplementary material for: Effects of the Toxic Non-Protein Amino Acid β-Methylamino-L-Alanine (BMAA) on Intracellular Amino Acid Levels in Neuroblastoma Cells
Source: Toxins (Basel). 2023 Nov 9;15(11):647. doi: 10.3390/toxins15110647 (PMC10674354; doi:10.3390/toxins15110647)
Supplement: Supplementary file 1 [file toxins-15-00647-s001.zip › toxins-2672170-supplementary.pdf]

Supplementary Materials: Effects of the Toxic Non-Protein Amino Acid β-Methylamino-L-Alanine (BMAA) on Intracellular Amino Acid Levels in Neuroblastoma Cells

Jake P. Violi, Lisa Pu, Sercan Pravadali-Cekic, David P. Bishop, Connor R. Phillips and Kenneth J. Rodgers

Table S1. Limit of Detection (LOD), Limit of Quantification (LOQ), and Multiple Reaction Monitoring (MRM) transitions for all amino acids analysed in this study. *denotes the internal standard.

Amino acid	Collision energy (eV)	MRM transitions (m/z)	LOD (ng/ mL)	LOQ (ng/mL)
Alanine	−29.40	131.10 → 44.15	0.02	0.05
	−8.80	→ 90.15		
Arginine	−22.00	175.20 → 70.15	1.89	5.72
	−12.00	→ 60.15		
Asparagine	−13.80	174.10 → 87.15	0.73	2.19
	−9.80	→ 133.10		
Aspartic acid	−22.10	175.10 → 74.10	0.98	2.99
	−9.20	→ 134.20		
BMAA	−17.00	119.10 → 44.10	0.38	1.17
	−15.00	→ 88.10		
Cysteine	−29.40	163.10 → 59.25	0.58	1.77
	−8.80	→ 122.15		
Glutamic acid	−16.00	148.10 → 84.10	1.49	4.51
	−21.90	189.10 → 84.10		
Glutamine	−20.00	147.10 → 84.10	0.47	1.44
	−14.00	→ 130.10		
Glycine	−17.90	117.20 → 76.20	3.14	9.52
	−7.20	→ 30.15		
Histidine	−15.00	156.15 → 110.15	4.80	14.55
	−28.00	→ 82.15		
Isoleucine	−15.40	173.20 → 86.25	0.03	0.09
	−8.80	→ 132.20		
Leucine	−15.40	173.20 → 86.25	0.02	0.06
	−8.80	→ 132.20		
Lysine	−14.00	147.20 → 67.10	2.86	8.67
	−30.00	→ 84.15		
Methionine	−30.30	191.10 → 61.20	0.19	0.58
	−7.80	→ 150.15		
Norvaline*	−16.90	159.10 → 72.05	0.02	0.06
	−8.20	→ 118.20		

Phenylalanine	−14.00	207.10 → 120.15	0.03	0.10
	−8.70	→ 166.20		
Proline	−24.10	157.00 → 70.10	0.02	0.05
	−9.80	→ 116.20		
Serine	−28.30	147.15 → 60.15	0.11	0.32
	−17.60	→ 42.15		
Threonine	−16.90	161.10 → 74.20	0.19	0.58
	−9.20	→ 120.20		
Tryptophan	−6.70	246.10 → 72.05	0.18	0.56
	−22.90	→ 118.20		
Tyrosine	−35.10	223.10 → 182.20	0.07	0.21
	−6.70	→ 91.05		
Valine	−16.90	159.10 → 72.05	0.05	0.16
	−27.30	→ 118.20		

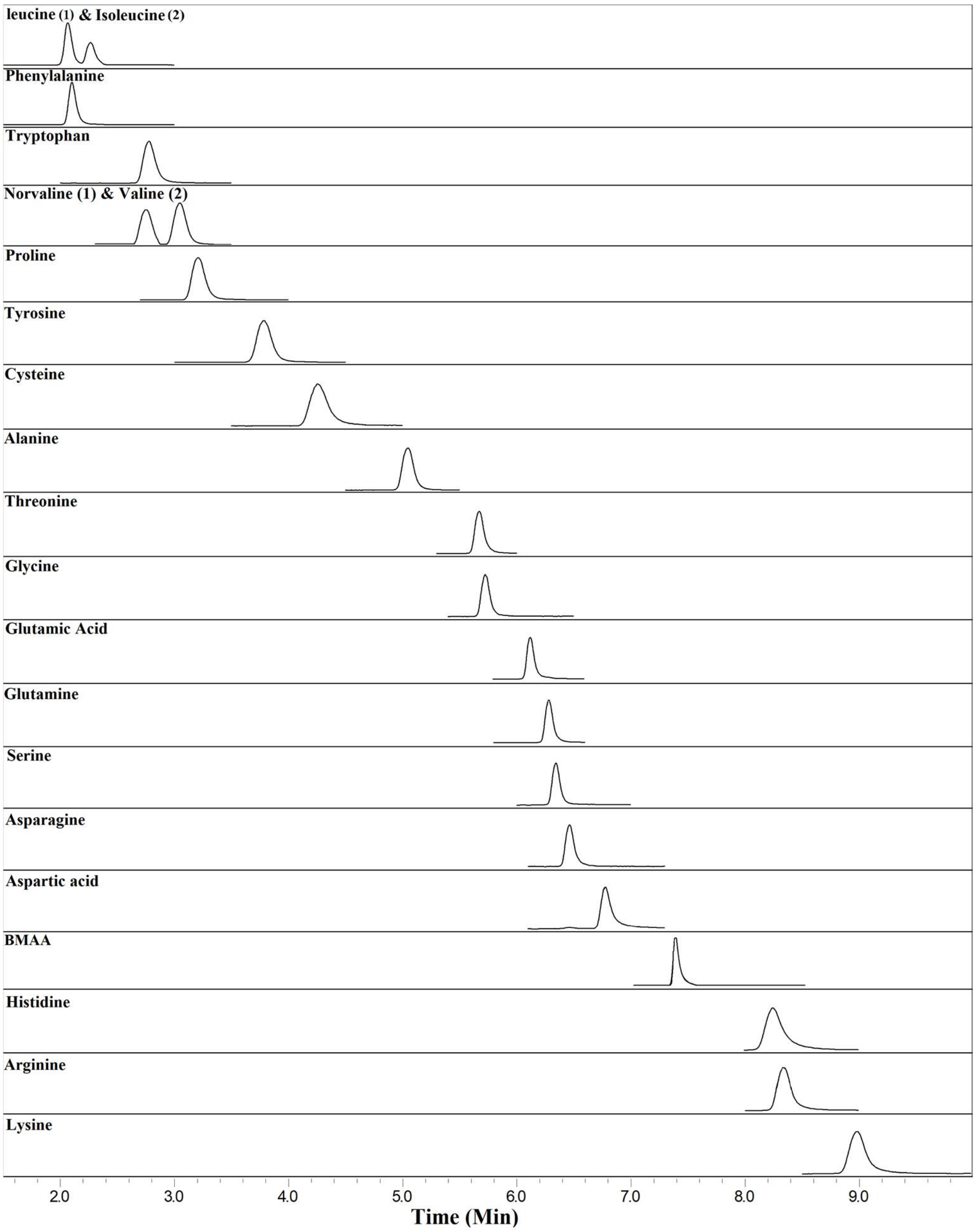

Figure S1. Total ion chromatograms (TICs) for each amino acid analysed.

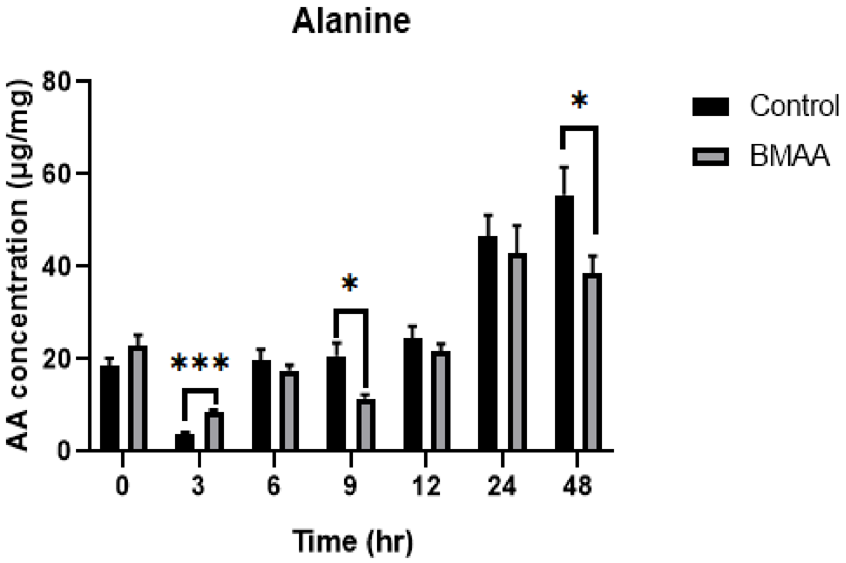

Figure S2. Intracellular concentrations of alanine for control and BMAA-treated cells (n = 6). Error bars denote the standard error of the mean (SEM). * Denotes a *p*-value of < 0.05, ** denotes a *p*-value of <0.005, and *** denotes a *p*-value of <0.0005.

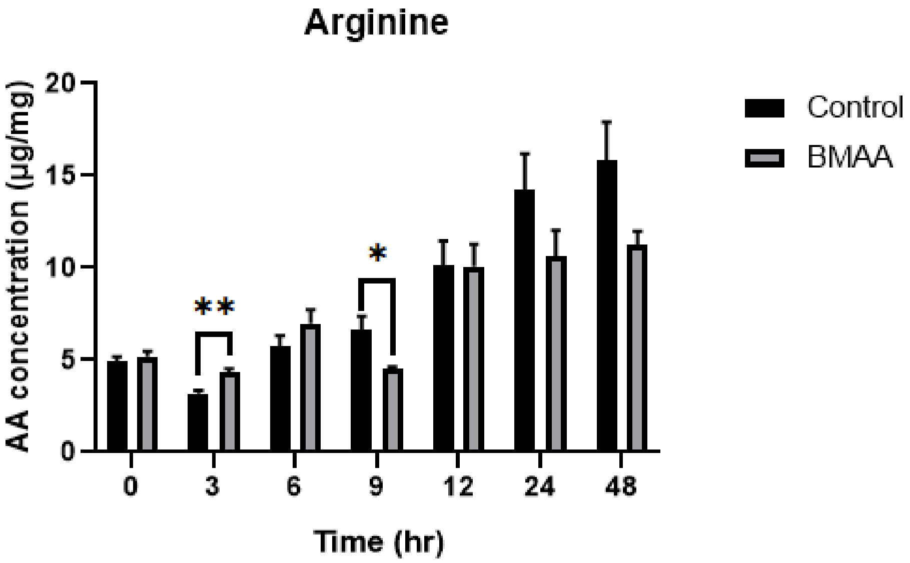

Figure S3. Intracellular concentrations of arginine for control and BMAA-treated cells (n = 6). Error bars denote the standard error of the mean (SEM). * Denotes a *p*-value of < 0.05 and ** denotes a *p*-value of <0.005.

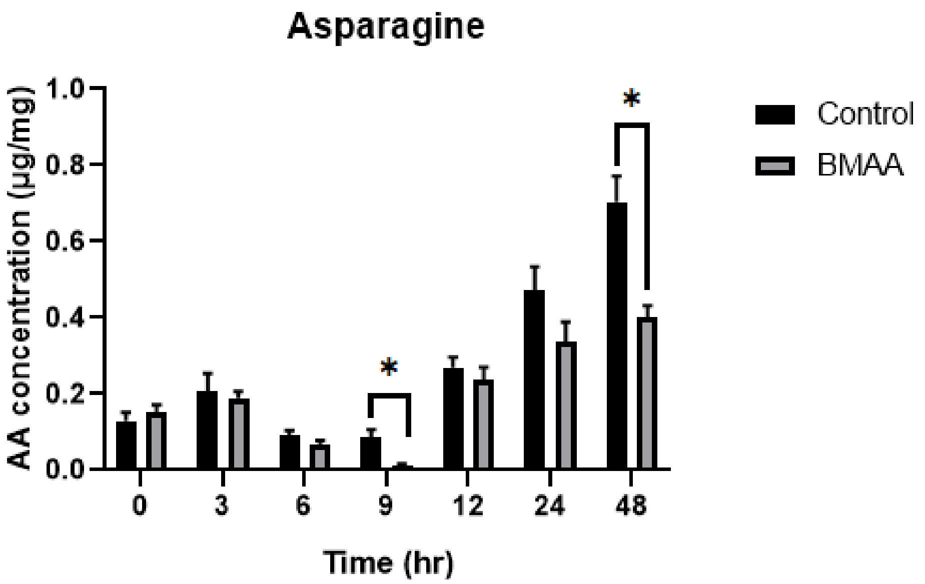

Figure S4. Intracellular concentrations of asparagine for control and BMAA-treated cells (n = 6). Error bars denote the standard error of the mean (SEM). * Denotes a *p*-value of < 0.05.

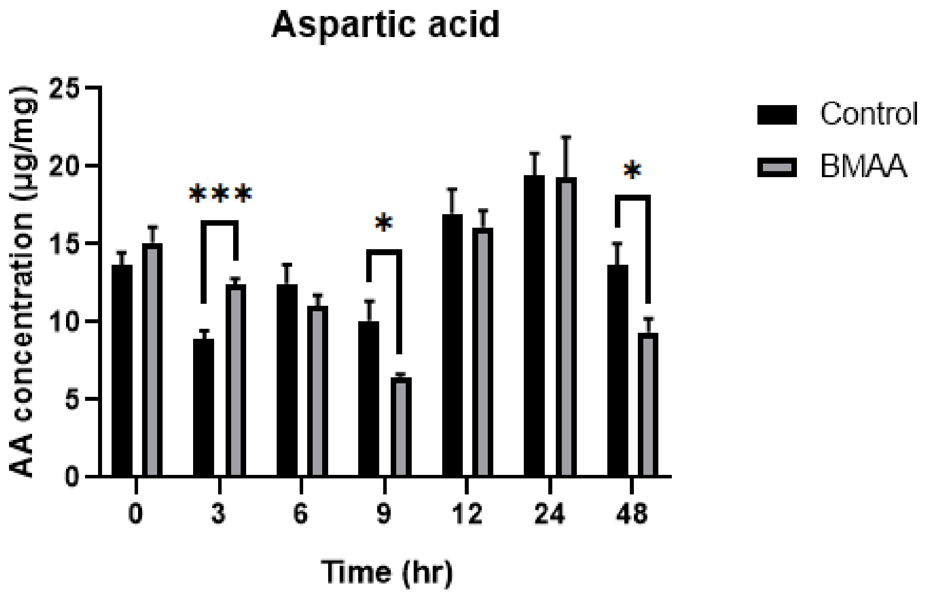

Figure S5. Intracellular concentrations of aspartic acid for control and BMAA-treated cells (n = 6). Error bars denote the standard error of the mean (SEM). * Denotes a *p*-value of < 0.05, ** denotes a *p*-value of <0.005, and *** denotes a *p*-value of <0.0005.

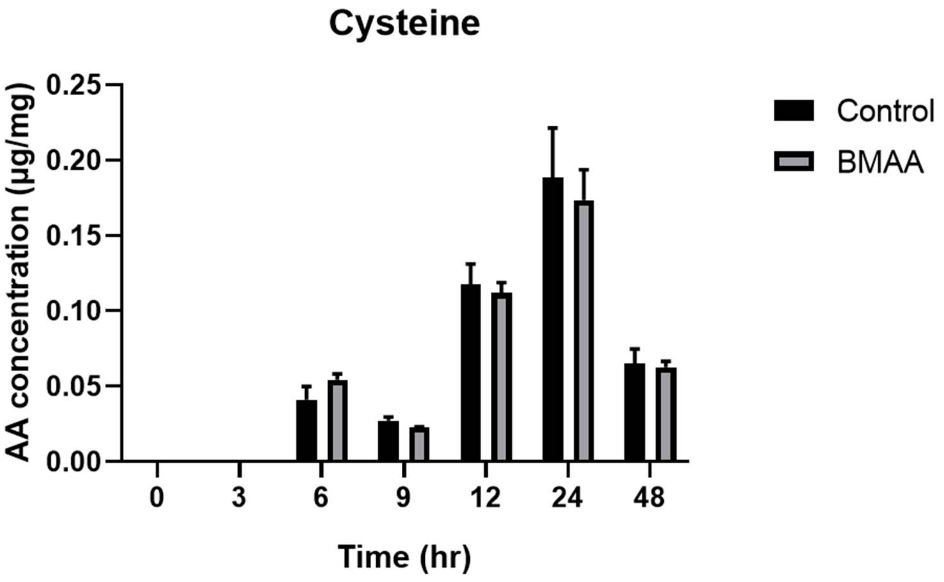

Figure S6. Intracellular concentrations of cysteine for control and BMAA-treated cells (n = 6). Error bars denote the standard error of the mean (SEM).

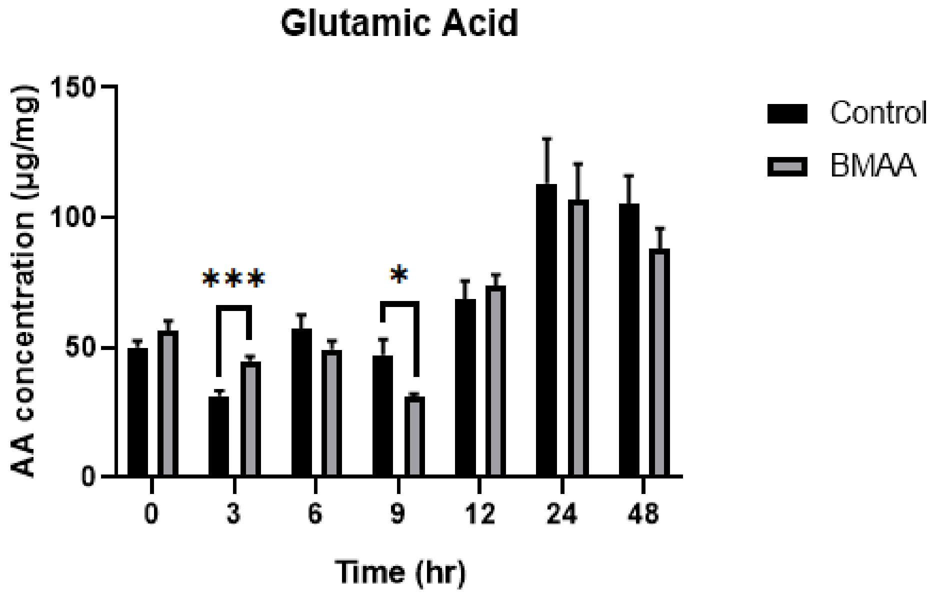

Figure S7. Intracellular concentrations of glutamic acid for control and BMAA-treated cells (n = 6). Error bars denote the standard error of the mean (SEM). * Denotes a p-value of < 0.05, ** denotes a p-value of <0.005, and *** denotes a p-value of <0.0005.

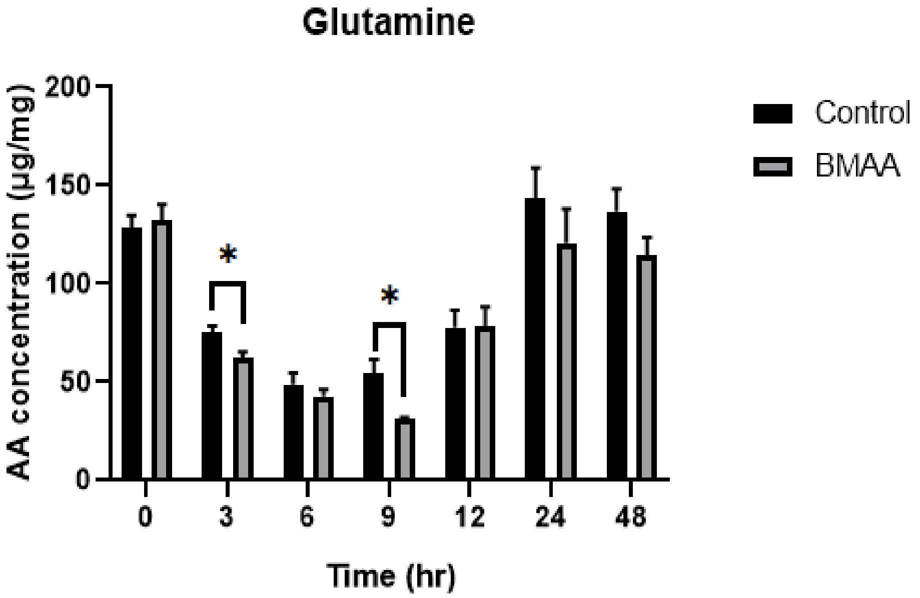

Figure S8. Intracellular concentrations of glutamine for control and BMAA-treated cells (n = 6). Error bars denote the standard error of the mean (SEM). * Denotes a *p*-value of < 0.05.

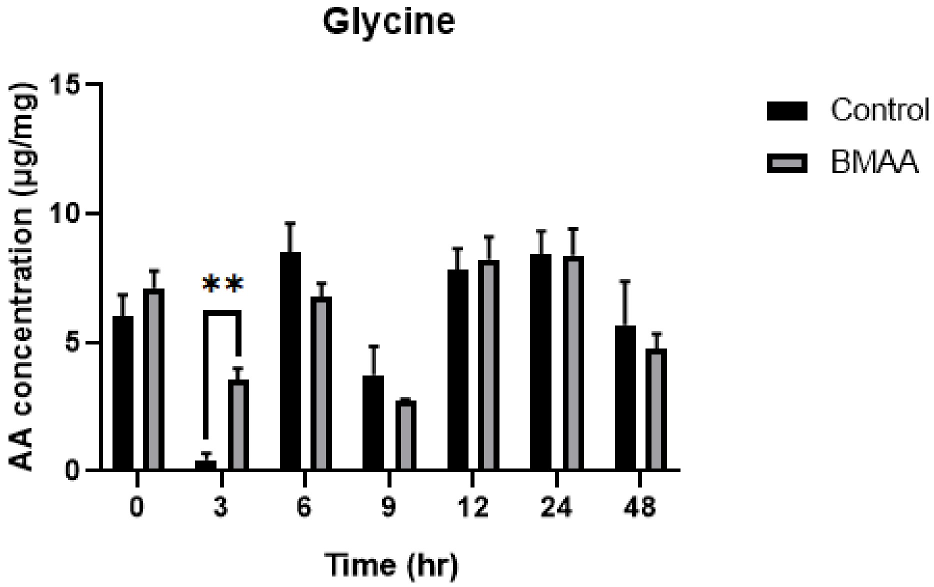

Figure S9. Intracellular concentrations of glycine for control and BMAA-treated cells (n = 6). Error bars denote the standard error of the mean (SEM). * Denotes a *p*-value of < 0.05 and ** denotes a *p*-value of <0.005.

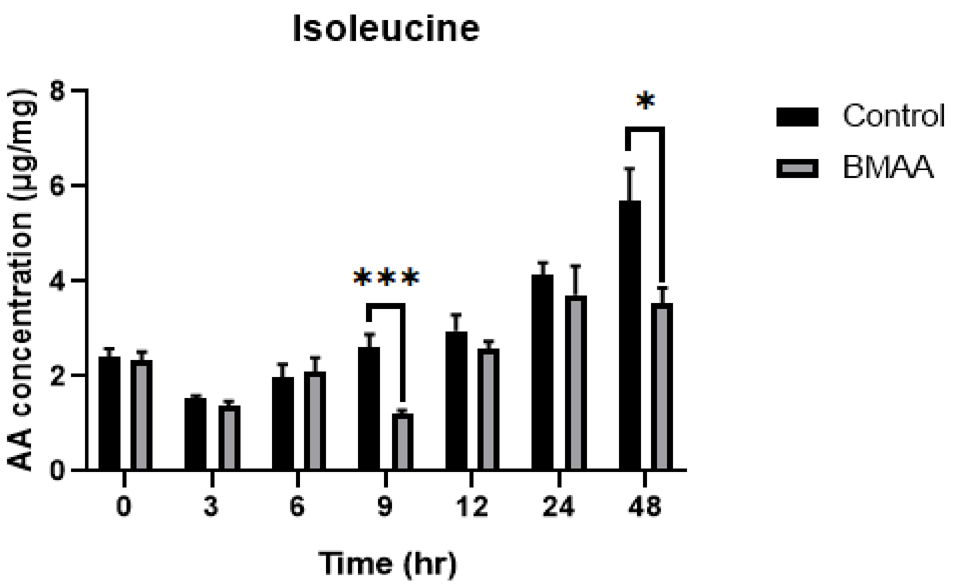

Figure S10. Intracellular concentrations of isoleucine for control and BMAA-treated cells (n = 6). Error bars denote the standard error of the mean (SEM). * Denotes a p-value of < 0.05, ** denotes a p-value of <0.005, and *** denotes a p-value of <0.0005.

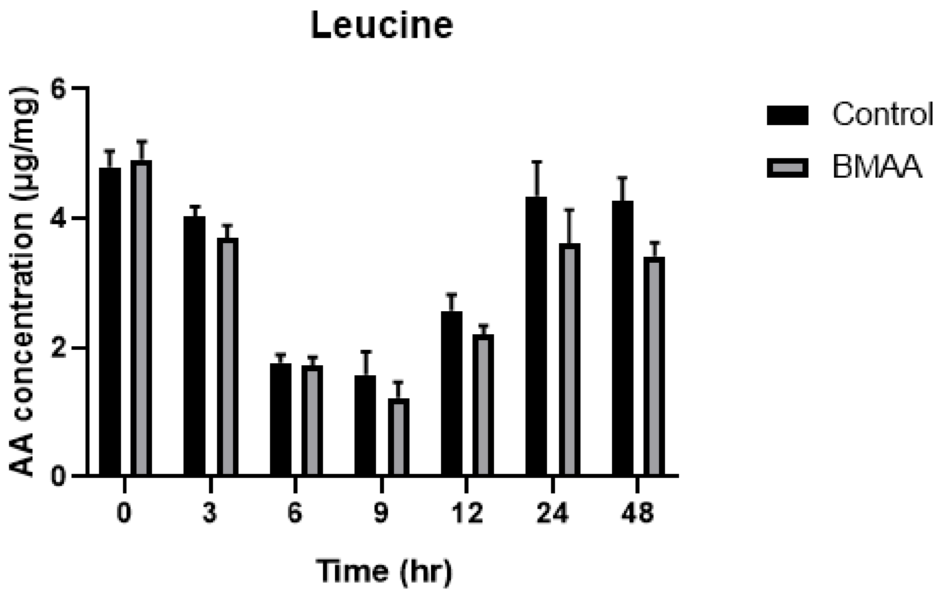

Figure S11. Intracellular concentrations of leucine for control and BMAA-treated cells (n = 6). Error bars denote the standard error of the mean (SEM).

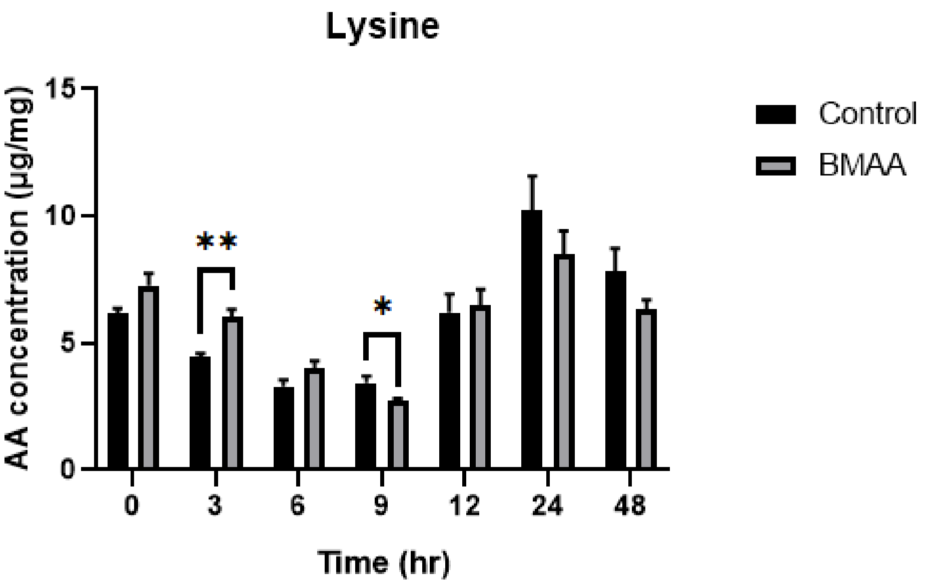

Figure S12. Intracellular concentrations of lysine for control and BMAA-treated cells (n = 6). Error bars denote the standard error of the mean (SEM). * Denotes a *p*-value of < 0.05 and ** denotes a *p*-value of <0.005.

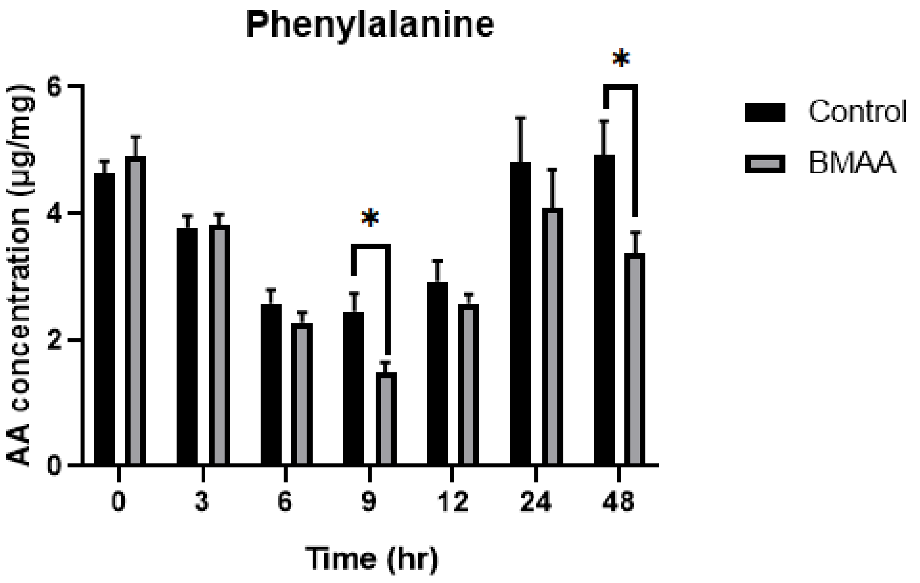

Figure S13. Intracellular concentrations of phenylalanine for control and BMAA-treated cells (n = 6). Error bars denote the standard error of the mean (SEM). * Denotes a *p*-value of < 0.05.

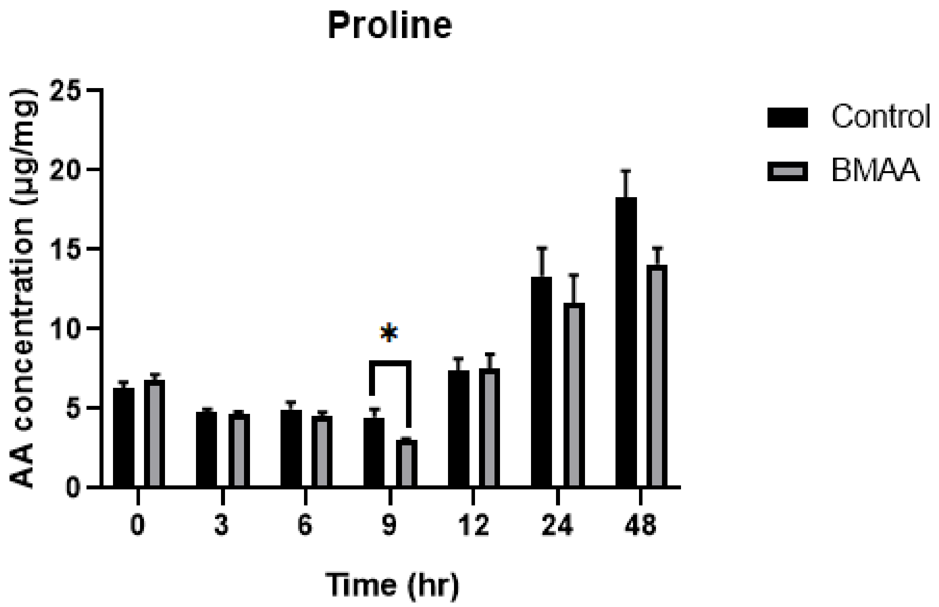

Figure S14. Intracellular concentrations of proline for control and BMAA-treated cells (n = 6). Error bars denote the standard error of the mean (SEM). * Denotes a *p*-value of < 0.05.

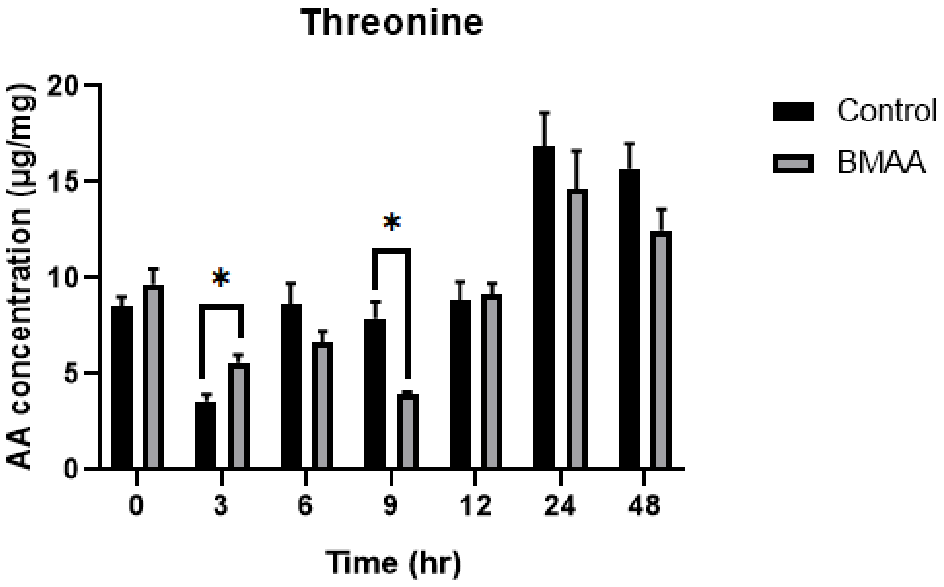

Figure S15. Intracellular concentrations of threonine for control and BMAA-treated cells (n = 6). Error bars denote the standard error of the mean (SEM). * Denotes a *p*-value of < 0.05.

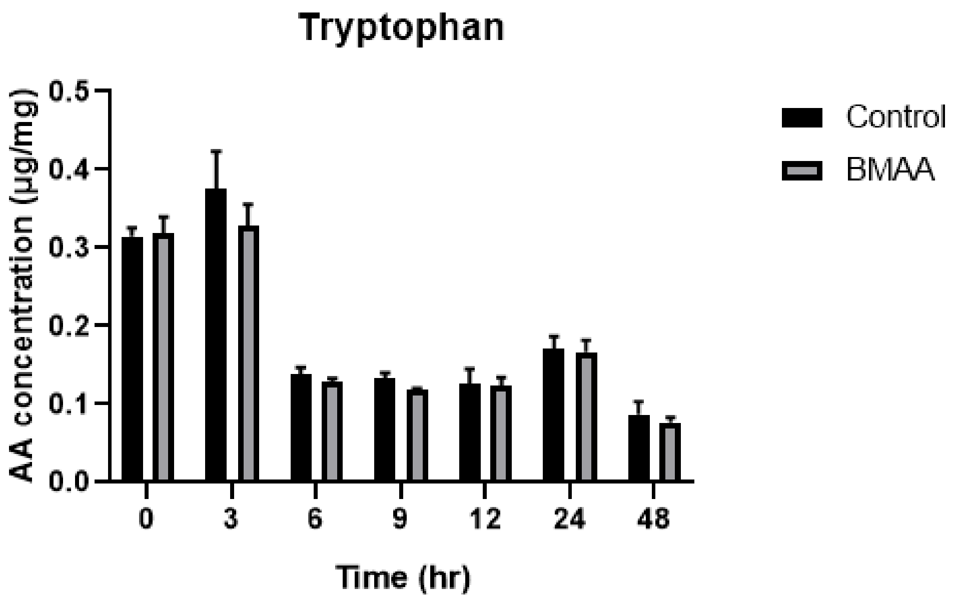

Figure S16. Intracellular concentrations of tryptophan for control and BMAA-treated cells (n = 6). Error bars denote the standard error of the mean (SEM).

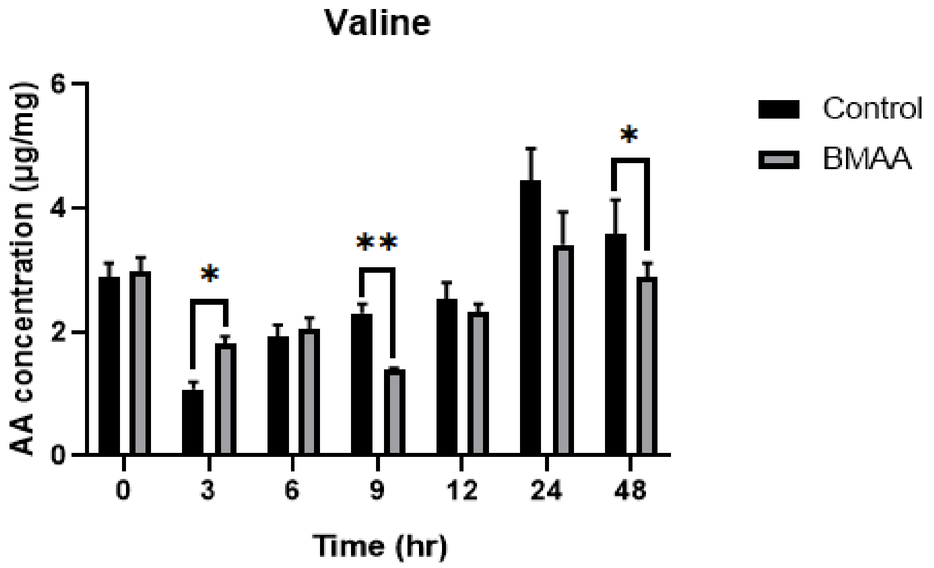

Figure S17. Intracellular concentrations of valine for control and BMAA-treated cells (n = 6). Error bars denote the standard error of the mean (SEM). * Denotes a p-value of < 0.05 and ** denotes a p-value of <0.005.

Table S2. Amino acid content of the culture medium used in the studies (ThermoFisher 11090081). *L-glutamine was freshly added before use from a 200 mM solution diluted 1 in 100.

**Amino acid**	**mg/L**	**mM**
L-Arginine hydrochloride	126	0.597
L-Cystine-2HCl	31	0.099
*L-Glutamine		2.0
L-Histidine hydrochloride-H2O	42	0.2
L-Isoleucine	52	0.397
L-Leucine	52	0.397
L-Lysine hydrochloride	73	0.397
L-Methionine	15	0.101
L-Phenylalanine	32	0.194
L-Threonine	48	0.403
L-Tryptophan	10	0.049
L-Tyrosine disodium salt dihydrate	52	0.199
L-Valine	46	0.393
